# Solid-State Nuclear Magnetic Resonance Insights into the Precursor-Dependent Structure and Na-Ion Storage Behavior of Na-Preintercalated Bilayered Vanadium Oxides

**DOI:** 10.1021/acs.chemmater.6c00065

**Published:** 2026-04-24

**Authors:** Xinle Zhang, Timofey Averianov, Mina Mozafari, Phillip Stallworth, Dmitri Barbash, Steven G. Greenbaum, Ekaterina Pomerantseva

**Affiliations:** † Department of Materials Science and Engineering, 6527 Drexel University, Philadelphia, Pennsylvania 19104, United States; ‡ Department of Physics & Astronomy, Hunter College of CUNY, New York, New York 10065, United States; § Materials Characterization Core Facilities, Drexel University, Philadelphia, Pennsylvania 19104, United States

## Abstract

Chemically preintercalated bilayered vanadium oxide (BVO) electrodes derived from V_2_CT_*x*_ MXene exhibit superior Na-ion storage performance compared to compositionally similar BVO counterparts synthesized from α-V_2_O_5_ powder. Here, we report for the first time the precursor-dependent structural differences in Na-preintercalated BVO electrodes (δ-Na_*x*_V_2_O_5_·*n*H_2_O) synthesized from α-V_2_O_5_ powder (AD-NVO) and V_2_CT_*x*_ MXene nanoflakes (MD-NVO) and show how these differences govern their electrochemical behavior in a nonaqueous Na-ion energy storage system. Our analyses show that AD-NVO and MD-NVO exhibit distinct compositions of δ-Na_0.37_V_2_O_5_·0.46H_2_O and δ-Na_0.33_V_2_O_5_·0.21H_2_O, respectively, along with pronounced differences in morphology, electronic structure, and interlayer chemistry. Scanning electron microscopy reveals the formation of 1D nanobelts for AD-NVO, whereas MD-NVO consists of 2D nanoflakes assembled into nanoflower-like agglomerates. X-ray photoelectron spectroscopy indicates that Na preintercalation led to different extents of V^5+^ to V^4+^ reduction in AD-NVO and MD-NVO, attributed to differences in the structural water content, which was further supported by the V^4+^ content quantification via electron paramagnetic resonance. Electrochemical measurements show fundamentally different charge storage behaviors: AD-NVO exhibits largely capacitive responses, while MD-NVO displays pronounced Na^+^ redox activity and delivers a higher specific capacity, improved rate capability, and superior cycling stability. Magic-angle spinning ^23^Na solid-state NMR identifies two distinct interlayer Na environments in MD-NVO, in contrast to a single Na site in AD-NVO. These sites play complementary roles, with one facilitating Na^+^ transport and the other acting as stabilizing pillars, as confirmed by ex situ X-ray diffraction. This study reveals how precursor-dependent structural evolution in chemically preintercalated layered oxides governs interlayer chemistry and electrochemical function, providing design principles for engineering layered metal oxides for advanced energy storage.

## Graphical abstract

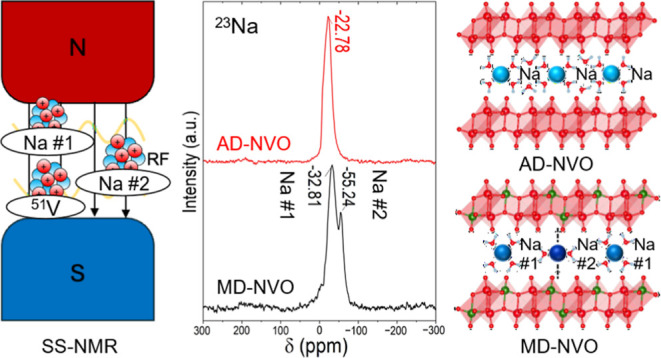

## Introduction

Sodium-ion batteries (SIBs) are attractive for large-scale energy storage where size and weight are less constrained, such as in the electrification of transport and electrical grid storage. Sodium, the fourth most abundant metallic element in the Earth’s crust,^1^ holds great potential to replace lithium in lithium-ion batteries (LIBs), thereby reducing dependence on strategic minerals and lowering manufacturing costs.^2,3^ SIBs share a similar intercalation mechanism with conventional LIBs, leading to extensive exploration of various intercalation-type cathode materials, including Prussian blue derivatives, polyanion compounds, and layered transition metal oxides (LTMOs).^4–7^ Specifically, layered electrode materials with two-dimensional (2D) structural channels typically offer more rapid ion diffusion, resulting in higher capacities.^7,8^ However, as the ionic radius of Na^+^ ions (1.02 Å) is larger than that of Li^+^ ions (0.76 Å), ion intercalation and diffusion in layered phases during battery operation can be hindered by narrow interlayer spacing, leading to limited capacities in SIBs. Therefore, cathode materials featuring expanded 2D ion transport channels are desirable to facilitate efficient Na^+^ ion intercalation and diffusion.

Layered vanadium oxides have shown great potential for beyond lithium-ion batteries due to the high redox activity of vanadium and 2D diffusion channels available for reversible ion intercalation/extraction. Bilayered vanadium oxide (BVO or δ-V_2_O_5_·*n*H_2_O) is characterized by a large interlayer spacing of 11.5 Å, stabilized by interlayer structural water molecules, making it a promising electrode material for beyond Li-ion intercalation and storage.^9,10^ BVO-based electrodes typically deliver high initial capacities due to the high oxidation state of vanadium, which can undergo multiple reduction steps accompanying intercalation of the electrochemically cycled ions. The ion diffusion process is further facilitated by the interlayer structural water that weakens the electrostatic interactions between electrolyte ions and the V–O layers.^11–13^ However, the high initial capacity of pristine BVO electrodes decays rapidly during Na^+^ ion cycling due to structural transformation or chemical degradation,^14^ limiting their potential in practical applications. Chemical preintercalation synthesis strategy can improve the Na-ion storage properties of LTMO electrodes by introducing the interlayer Na-ion sites to the BVO structure before electrochemical cycling. The chemical preintercalation synthesis of BVOs is based on a well-known sol–gel or precipitation reaction, which involves the dissolution and recrystallization of the vanadium-containing precursor (α-V_2_O_5_ powder or V_2_CT_*x*_ MXene nanoflakes) in an aqueous medium.^15^ In this process, water-soluble sodium-containing salt dissociates into cations and anions, providing atomic-scale homogeneous distribution in solution and allowing the cations to be uniformly trapped within the interlayer regions of BVO during the subsequent gelation or precipitation step.^16^ It has been demonstrated that the chemical preintercalation of inorganic metal ions creates the ion diffusion sites and predefines the ion diffusion pathways for BVO electrodes before the electrochemical cycling, thereby improving the specific capacity or/and cycling stability.^17–21^ Therefore, for SIBs, the Na^+^ ion preintercalated BVO (NVO) electrodes have been shown to have improved Na-ion storage capacity.^13,22^ The first NVO electrode material was reported in 2016, synthesized using the α-V_2_O_5_ precursor with prolonged aging and a hydrothermal treatment step, which led to the formation of single-phase one-dimensional (1D) nanobelts with high crystallinity, and demonstrated high initial specific capacity of 365 mAh g^–1^ at 20 mA g^–1^ within the potential window of 1.0–4.3 V in Na-ion cells. Later, the interlayer structural water content in the NVO nanobelts has demonstrated its tunability enabled by vacuum annealing treatment, and it has been reported that the additional annealing step effectively reduced the hydration degree (*n* in δ-Na_*x*_V_2_O_5_·*n*H_2_O) from 1.2 to 0.5, and introduced additional bonds connecting the V–O bilayers through the interlayer Na sites that enhanced the cycling stability in nonaqueous Na-ion cells. The chemical preintercalation of inorganic metal ions into the BVO structure has been later achieved by using V_2_CT_*x*_ MXene as the synthesis precursor.^19^ V_2_CT_*x*_ MXene-derived NVO (MD-NVO) and α-V_2_O_5_ derived NVO (AD-NVO) show similar X-ray diffraction (XRD) patterns and interlayer distances of ∼10.80 Å, but instead of 1D nanobelts, the MD-NVO shows a unique 2D nanoflake morphology with nanoflower-shaped particles.^19,23^ While the nonaqueous Na-ion cycling performance of the MD-NVO electrode is reported for the first time in this work, in nonaqueous Li-ion cells, it has been demonstrated that the V_2_CT_*x*_ MXene precursor enables an improved electrochemical stability compared to the α-V_2_O_5_ precursor.^16^ The superior electrochemical stability was attributed to the unique 2D particle shape of the V_2_CT_*x*_-derived BVOs enabling the morphological stabilization effect. However, no further mechanistic insights, specifically structural characteristics beyond interlayer distances, have been provided to support and correlate with the enhanced nonaqueous cycling stability observed for the V_2_CT_*x*_-derived chemically preintercalated BVOs.

Solid-state nuclear magnetic resonance (ss-NMR) spectroscopy, probing the nuclei of the electrode material constituting elements, has been employed in revealing the local structure and chemical environments of the vanadium oxide polymorphs.^24,25^ For example, ^51^V and ^17^O ss-NMR were utilized to investigate the local structural evolution in V_2_O_5_·*n*H_2_O polymorphs during the dehydration process. The results of this study revealed that multiple ^51^V resonances ranging from −580 ppm to −663 ppm in hydrated V_2_O_5_ were merged into a single peak at −620 ppm when fully dehydrated V_2_O_5_ was formed, corresponding to the bilayered to monolayered structural transformation.^26^ ^7^Li and ^23^Na ss-NMR can be respectively utilized to study the local Li or Na sites in battery active materials or electrodes, assisting in elucidating the mechanisms of materials synthesis or electrochemical charge storage processes. For instance, ^7^Li NMR has been used to distinguish the lithium sites in the prelithiated δ-Li_*x*_V_2_O_5_ and γ-Li_*x*_V_2_O_5_ polymorphs, and the lithium quantification in each phase was achieved via the electron paramagnetic resonance (EPR) analysis.^25,27^ Later, three distinct ^7^Li NMR peaks have been identified in the ss-NMR spectrum of Li_*x*_V_2_O_5_ electrode cycled in Li-ion cells using an ex situ method, distinguishing the Li^+^ ions from the residue electrolyte, solid electrolyte interface on the surface of the electrode, and those intercalated and diffused into the Li_*x*_V_2_O_5_ structure.^28^ As interest in sodium-ion chemistry in batteries rises, ^23^Na NMR has been utilized to understand the Na^+^ ion intercalation and diffusion mechanism in the electrodes for Na-ion cells.^29,30^ However, due to the lack of knowledge about the interlayer region structure, the mechanism of electrochemical stability improvement demonstrated by chemically preintercalated BVO electrodes remains unclear. Moreover, no study has directly compared the differences between the chemically preintercalated BVO active materials obtained from α-V_2_O_5_ and V_2_CT_*x*_ MXene precursors beyond their particle morphologies, and no specific structural characteristics and structure–property relationships have been established for the chemically preintercalated BVOs.

In this work, ss-NMR characterization of the active materials allowed us, for the first time, to identify two distinct Na sites in the MD-NVO structure, whereas only a single Na site was observed in AD-NVO, establishing a correlation between chemically preintercalated Na sites and the charge storage behavior in nonaqueous Na-ion cells. Both materials showed interlayer distances of 10.70–10.95 Å, as suggested by XRD, while their chemical compositions, determined by atomic absorption spectroscopy (AAS) and thermogravimetric analysis, revealed varying preintercalated Na and interlayer water content in AD-NVO (Na_0.37_V_2_O_5_·0.46H_2_O) and MD-NVO (Na_0.33_V_2_O_5_·0.21H_2_O). We confirmed the 1D nanobelt morphology for AD-NVO and nanoflower-shaped particles that are composed of 2D nanoflakes for MD-NVO via scanning electron microscopy (SEM). Through X-ray photoelectron spectroscopy (XPS), we identified a significantly higher V^4+^/V^5+^ ratio in MD-NVO (0.39) compared to AD-NVO (0.04). This finding was further supported by the EPR analysis, which revealed nearly twice the V^4+^ spin count in MD-NVO relative to AD-NVO, confirming the higher fraction of reduced V^4+^ enabled by the V_2_CT_*x*_ MXene precursor. Here, we for the first time report the nonaqueous charge storage properties of MD-NVO electrode in Na-ion cells with the reference of the AD-NVO electrode using cyclic voltammetry (CV), galvanostatic cycling, and rate capability experiments. The electrochemical characterization results revealed substantial differences in the nonaqueous Na-ion cycling performance of the AD-NVO and MD-NVO electrodes, which strongly correlated with their distinct local interlayer Na^+^-H_2_O coordination identified by ^23^Na ss-NMR. This work reveals how precursor-enabled interlayer metal-ion sites influence nonaqueous cycling performance and provides insight into general mechanisms that may enhance the performance of other chemically preintercalated layered metal oxides.

## Experimental Methods

### Synthesis of α-V_2_O_5_-Derived Na_*x*_V_2_O_5_·*n*H_2_O (AD-NVO)

The synthesis procedure of AD-NVO was adapted from the previous report, as illustrated in Figure S1a, Supporting Information.^17^ 1.61 g NaCl (Fisher Scientific, USA) was dissolved in 15 mL of deionized water (Fisher Scientific, USA) in a 250 mL beaker, followed by addition of 15 mL of 30 wt % hydrogen peroxide (Fisher Scientific, USA) under stirring. 0.5 g of α-V_2_O_5_ powder (Alfa Aesar, USA) was slowly added to the beaker over a span of 15 min under stirring at 500 rpm, leading to the mixture color change from bright yellow to transparent orange hue. The mixture was then stirred at room temperature for 1 h, followed by the temperature increase to 60 °C and stirring at this temperature for 4 h. The beaker was covered with Parafilm while stirring at 60 °C to prevent excessive water evaporation. After 4 h, the produced dark brown precipitate was aged for 4 days. The precipitate was then transferred to a Teflon-lined stainless-steel autoclave (Parr Instrument, USA) and mixed with 15 mL of a fresh 0.5 M NaCl solution followed by hydrothermal treatment at 220 °C for 24 h. The solid material, AD-NVO, after hydrothermal treatment, was thoroughly washed and filtered using deionized water and air-dried at 105 °C in air overnight. The α-V_2_O_5_ to AD-NVO transformation is schematically illustrated in Figure 1a.

**Figure 1:**
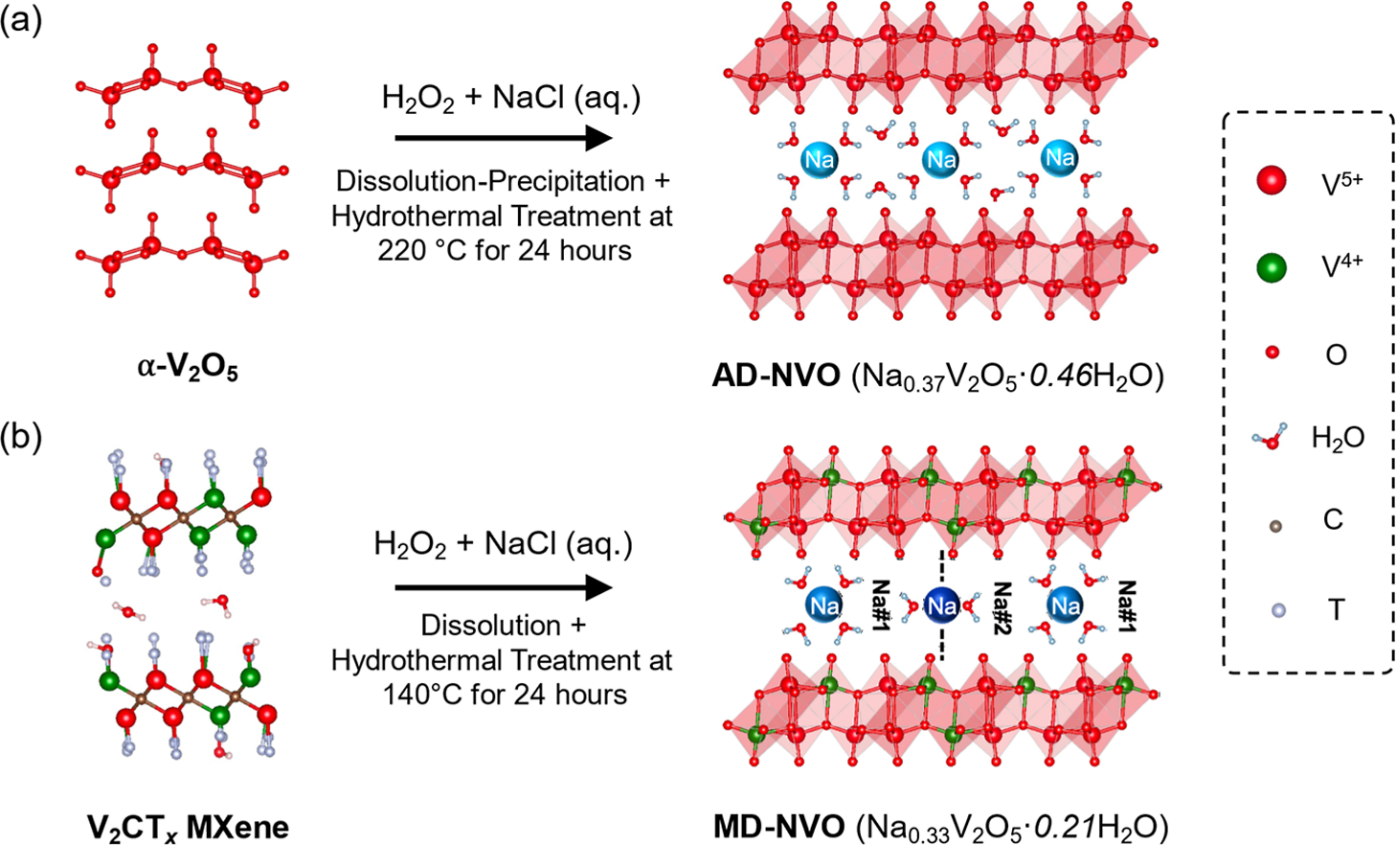
Illustrations of the structure reconstruction of (a) α-V_2_O_5_ to AD-NVO and (b) V_2_CT_*x*_ MXene to MD-NVO in chemical preintercalation synthesis.

### Synthesis of V_2_CT_*x*_ MXene-Derived Na_*x*_V_2_O_5_·*n*H_2_O (MD-NVO)

The V_2_CT_*x*_ MXene precursor was synthesized according to the established diluted mixed acid etching protocol for the V_2_AlC MAX phase.^19,31,32^ The synthesis of MXene-derived δ-Na_*x*_V_2_O_5_·*n*H_2_O (MD-NVO) was adapted from a previous report, as shown in Figure S1b, Supporting Information.^33^ 300 mg of multilayer V_2_CT_*x*_ powder was added to 30 mL of a 0.5 M NaCl (Fisher Scientific, USA) solution in a 125 mL flask under stirring at 300 rpm, followed by addition of 2 mL of 30 wt % hydrogen peroxide (H_2_O_2_, Fisher Scientific) and stirring for 2 h. Stirring was then turned off, and the solution sat to settle for 30 min to enable an effective separation of the supernatant solution from unreacted V_2_CT_*x*_ MXene nanoflakes. The resulting dark-green supernatant solution was transferred into a Teflon-lined stainless-steel autoclave (Parr Instrument, USA) for hydrothermal treatment at 140 °C for 24 h. After cooling, the crystallized precipitate was dispersed in 200 mL of deionized water, vacuum filtered, thoroughly washed, and dried at 105 °C in air overnight. The V_2_CT_*x*_ MXene to MD-NVO transformation is schematically illustrated in Figure 1b.

### Materials Characterizations

XRD measurements were conducted using a Rigaku MiniFlex 600 with Cu Kα radiation (λ = 1.54 Å) in a 2θ range from 3° to 60° with a step size of 0.02° and step duration of 0.5 s. The *d*-spacings calculation and background subtractions for XRD data sets were performed using CrystalDiffract 7 software. Thermogravimetric analysis (TGA) experiments were performed using a Q50 thermogravimetric analyzer (TA Instruments, USA) in the temperature range from 25 to 1000 °C with a ramping up rate of 10 °C min^–1^. The temperature was held at 100 °C for 60 min to ensure that all physisorbed water was removed prior to evaluating the weight loss corresponding to the removal of structural water; the analysis was performed using the TGA data above 100 °C. The SEM images were captured using Apreo 2S Low Vac (ThermoFisher, USA) equipped with a trinity in-lens detector with a 5 kV electron accelerating voltage. The sodium and vanadium contents were determined using a flame atomic absorption spectrometer (AAS) (Shimadzu AA7000, Japan) equipped with an air-acetylene or nitroxide-acetylene burner. The sodium and vanadium calibration curves were developed using standard solutions prepared from 1000 ppm stock solutions (Inorganic Ventures, USA). For AAS sample preparation, approximately 10 mg of MD-NVO or AD-NVO powder was dispersed in a 50 mL beaker containing analytical-grade water, followed by adding one drop of 30 wt % H_2_O_2_ (Fisher Scientific, USA) to aid dissolution. The mixture was bath-sonicated for 10 min, then transferred and graduated to a 100 mL volumetric flask using analytical-grade water. 20 mL of the solution was transferred and graduated to another 100 mL volumetric flask to obtain the final AAS sample. The XPS spectra were recorded on a PHI VersaProbe 5000 (Physical Electronics, USA) using a monochromatic Al Kα source and ionized Ar for charge compensation. The high-resolution V 2p spectra were acquired at a pass energy of 27.0 eV with a step size of 0.050 eV, and the Na 1s spectra were acquired at the same pass energy with a step size of 0.025 eV. The peak fitting and data analysis were carried out using CasaXPS software. A Shirley background was applied for the V 2p spectra quantification.

### Solid-State NMR and EPR Measurements

A Varian-Agilent NMR spectrometer operating at 300 MHz (^1^H frequency) was used for ^7^Li and ^23^Na solid-state NMR magic angle spinning (MAS) experiments with 3.2 mm rotors. The spin rates were set at 17, 18, 20, and 20 kHz for the measurements of AD-NVO, MD-NVO, AD-LVO, and MD-LVO, respectively. Reference samples for Li and Na for both chemical shift and intensity calibration were aqueous solutions of LiCl and NaCl, respectively. The MAS NMR measurements were conducted at around 117 MHz for ^7^Li and 79 MHz for ^23^Na. ^51^V solid-state MAS NMR experiments were performed using a Bruker Avance 600 MHz spectrometer with 1.2 mm rotors and spinning at 55 kHz with a single-pulse sequence and 2 μs 90° pulses with repetition delay of 1s. Aqueous NaVO_3_ was used as the ^51^V shift reference. All NMR measurements were conducted at 20 °C. EPR measurements were carried out at 20 °C by using a Bruker EPR X-band benchtop instrument (ESR5000). V_2_O_4_ was used as a standard for spin-counting in the EPR measurements. Integrated intensities were obtained by double integration of the EPR derivative spectra.

### Electrochemical Characterization

The AD-NVO and MD-NVO powders were vacuum-dried at 200 °C in glass vials in an oven for 48 h prior to the electrode fabrication procedures for further dehydration, a proven approach to enhance the cycling stability of chemically preintercalated bilayered vanadium oxides in cells with nonaqueous electrolytes.^17,34^ The slurries for electrode fabrication were prepared by mixing active material, carbon black (Thermo Scientific Chemicals, USA), and Kynar Flex PVDF (Arkema, USA) with a gravimetric ratio of 7:2:1 in a polypropylene cup containing 1-methyl-2-pyrrolidinone (Acros Organics, USA). Flacktek SpeedMixer (Flacktek, USA) was used to assist the mixing of the slurries, which were then coated onto the aluminum foil current collectors using an 80 μm height doctor blade. The wet electrode films were air-dried in a fume hood overnight, followed by being vacuum-dried at 105 °C for 24 h. Then, the dried electrode films were punched into 10 mm electrode disks, which were further vacuum-dried at 150 °C for 24 h before being transferred to an Ar-filled glovebox. The electrode disks were assembled into type 2032 coin cells for electrochemical testing. For each cell, 1 M NaClO_4_ in PC/EC/FEC (50/45/5 vol %) served as an electrolyte, metallic Na chips served as both counter and reference electrodes, and glass microfiber filters (Whatman, UK) were used as a separator. The CV experiments were conducted using a multichannel potentiostat (Biologic VMP3, USA) at a scan rate of 0.1 mV s^–1^. The cycle life and rate capability of the cells were evaluated by performing galvanostatic cycling using a multichannel electrochemical workstation (Arbin Instrument, USA). A 20 mA g^–1^ current density was applied for cycle life evaluation; the stepwise increased current densities of 20, 50, 100, and 200 mA g^–1^ were applied for each 10 galvanostatic cycles for the rate capability test, and then, the current density was brought back to 20 mA g^–1^ for an additional 10 cycles to evaluate the capacity recoverability after high-rate operation. All electrochemical testing experiments were conducted at room temperature and within a potential window of 1.2–3.5 V vs Na/Na^+^. All potentials in this work are reported with respect to the Na/Na^+^ reference electrode.

## Results and Discussion

The XRD patterns, shown in Figure 2a, demonstrate the intense (001) reflections with a few Bragg peaks at higher 2θ values for both samples, consistent with the nanostructured BVO phase reported in the literature.^19,23^ The (001) peaks for AD-NVO and MD-NVO are positioned at 2θ of 8.06° and 8.26°, corresponding to the *d*-spacings of 10.95 and 10.70 Å, respectively. These structural parameters agree with the previously reported structures for the BVO polymorphs containing hydrated metal-ions in the interlayer region (JCPDS #88-0579).^35^ Additionally, the (001) peaks in the XRD patterns exhibit fwhm values of 0.73° 2θ for AD-NVO and 0.51° 2θ for MD-NVO, indicating a higher crystallinity of MD-NVO compared to that of AD-NVO.

**Figure 2:**
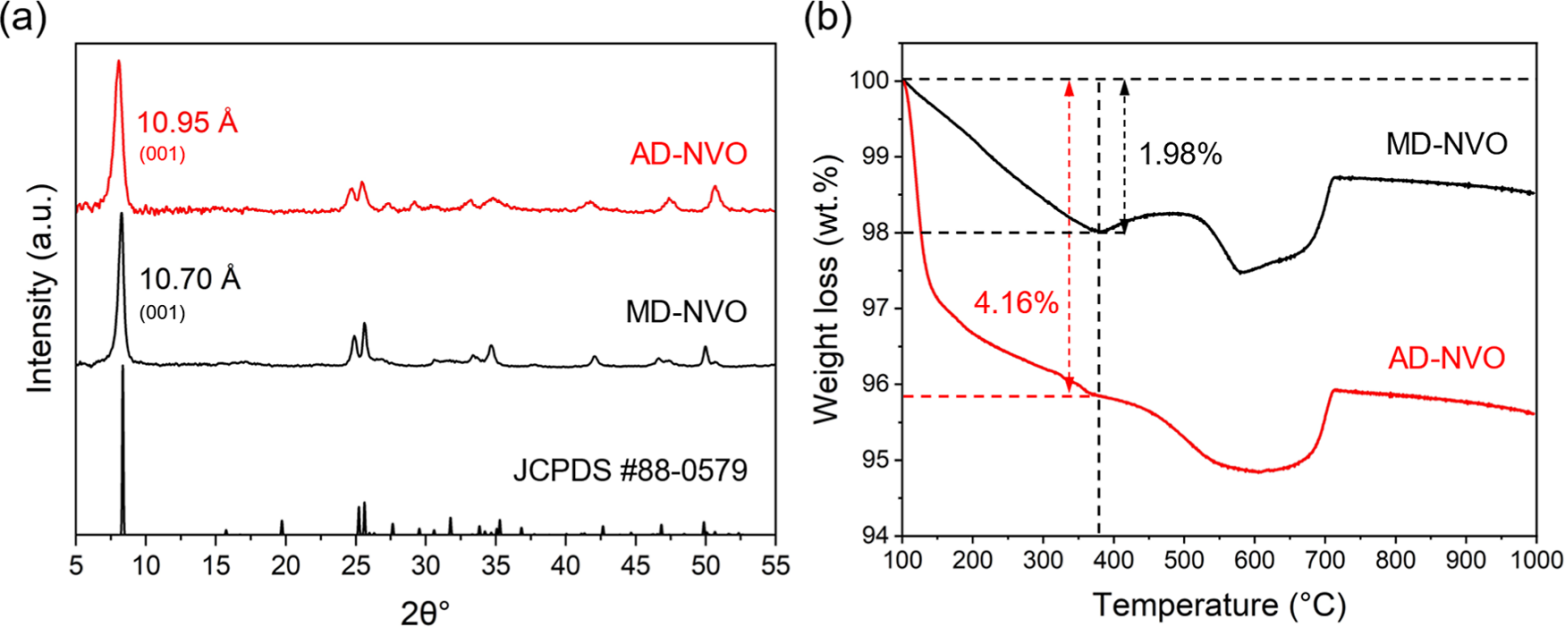
(a) XRD patterns and (b) TGA weight loss curves of AD-NVO and MD-NVO.

TGA experiments were combined with AAS analysis to determine the chemical compositions of both materials. The weight loss between 100 and 375 °C (Figure 2b) is attributed to the structural water loss from the interlayer regions.^22^ The analysis of TGA data suggests the 1.98% weight loss for MD-NVO and 4.16% weight loss for AD-NVO, indicating the structural water content in AD-NVO is more than double that in MD-NVO. The concentrations of Na and V in each sample versus the corresponding calibration curves, determined using AAS, are shown in Figure S2 and in the Supporting Information. The AAS and TGA analyses results are summarized in Table 1, suggesting the chemical formulas of Na_0.37_V_2_O_5_·0.46H_2_O for AD-NVO and Na_0.33_V_2_O_5_·0.21H_2_O for MD-NVO. Interestingly, while a similar degree of Na preintercalation was determined for both materials (0.37 for AD-NVO and 0.33 for MD-NVO), a substantial difference in structural water content was observed, indicating the discrepancies in hydration degrees of interlayer Na^+^ ions. This finding indicates that the synthesis precursor and routes can impact the chemical compositions of the interlayer region of the BVO polymorphs.

**Table 1:** Summary of the AAS and TGA Analyses for AD-NVO and MD-NVO, and Corresponding Chemical Formulas of the Materials under Study

samples	Na/V ratio (via AAS)	weight loss (wt %, via TGA)	chemical formula
AD-NVO	0.1860	4.16	Na_0.37_V_2_O_5_·0.46H_2_O
MD-NVO	0.1668	1.98	Na_0.33_V_2_O_5_·0.21H_2_O

SEM images (Figure 3) show the morphological differences enabled by the AD and MD synthesis routes. As Figure 3a demonstrates, the AD-NVO crystallized as nanobelts, and these 1D nanoparticles entangled to form highly porous nest-like agglomerates. The SEM images of AD-NVO particles, shown in Figure 3c, suggest that the width of each nanobelt is between 50 and 100 nm and the length is over 1 μm, in agreement with the literature. The morphology of MD-NVO (Figure 3d) exhibits nanoflower-like particles that are composed of 2D nanoflakes in agreement with the previous report.^19^ The size of the individual nanoflake is about 1–3 μm in lateral dimension and 50–80 nm in thickness. It is believed that compared to AD-NVO nanobelts, the 2D nanoflakes of MD-NVO can suppress the active material degradation during the Na-ion cycling, attributed to a 2D-enhanced morphological stabilization effect.^36^ The larger 2D nanoflakes are associated with a reduced relative surface area, which can suppress irreversible electrochemical processes during prolonged cycling, such as the extraction of pillaring ions and vanadium oxide dissolution.^14,22,37^ The detailed mechanistic insights into morphological stabilization are beyond the scope of this study and will be the subject of future work.

**Figure 3:**
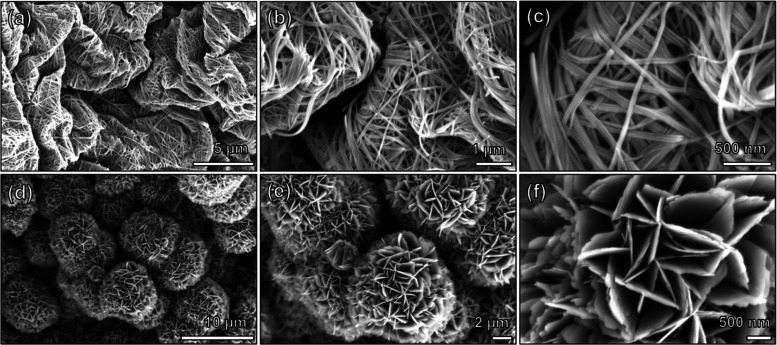
SEM images showing (a–c) AD-NVO nanobelts and (d–f) MD-NVO nanoflakes assembled into nanoflower-like particles captured at (a,d) low magnification, (b,e) medium magnification, and (c,f) high magnification.

Figure 4 demonstrates the XPS characterization and analysis of AD-NVO and MD-NVO, with the corresponding survey spectra (Figure S3 in the Supporting Information) confirming the presence of Na, V, and O. The high-resolution V 2p spectra shown in Figure 4a,b indicate that the peaks at binding energies of 517.2 and 524.5 eV correspond to V 2p_3/2_ and V 2p_1/2_ features of V^5+^, respectively.^38^ The V^4+^ oxidation state also presented in both samples at different levels, as suggested by the V 2p_3/2_ and V 2p_1/2_ peaks of V^4+^ at 516.0 and 523.3 eV, respectively.^38^ The XPS spectra analysis of the V 2p region, summarized in Table 2, indicates that MD-NVO contains 28.2% of V^4+^, which is substantially higher than the 4.25% V^4+^ content in AD-NVO. This observation drew our attention to revisit the chemical formula of both materials, which were characterized by a similar degree of Na preintercalation but exhibited a substantial variation in structural water content. The discrepancy in vanadium oxidation states in both materials suggests that the interlayer Na^+^ ions and structural water may be coordinated differently in the interlayer regions of AD-NVO and MD-NVO, resulting in distinct bonding environments and intercalation sites that lead to different extents of vanadium reduction. Figure 4c shows that the Na 1s signal of AD-NVO consists of a clear single peak at 1071.5 eV. In contrast, the Na 1s characteristic in the MD-NVO spectrum appears to separate into two peaks at 1071.4 and 1071.7 eV, with a splitting of 0.3 eV (Figure 4d), indicating the possibility for the presence of two Na sites in the interlayer region. Additionally, the formation of the larger fraction of V^4+^ in MD-NVO can be related to the enhanced electrostatic interactions between the interlayer Na^+^ ions and the V–O layers due to a lower interlayer hydration degree, as compared to AD-NVO, thereby potentially improving the structural stability due to the formation of new bonds in the interlayer regions.^13,22^

**Figure 4:**
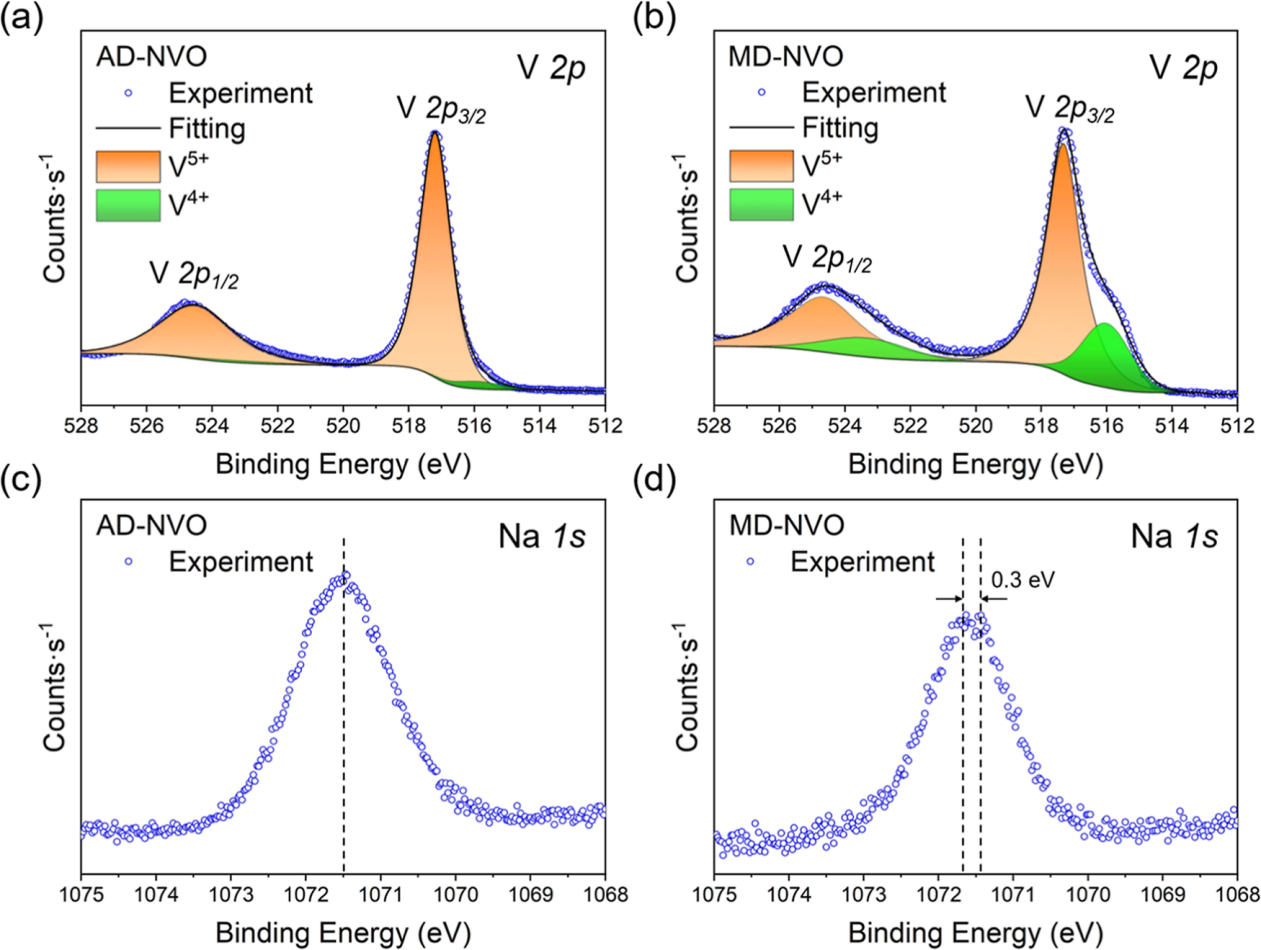
XPS spectra in the (a,b) V 2p region and (c,d) Na 1s region of (a,c) AD-NVO and (b,d) MD-NVO.

**Table 2:** Summary of the EPR Analysis of AD-NVO and MD-NVO

sample	resonance (*B*_0_/mT)	max intensity	number of V^4+^ Spin
MD-NVO	341.74	2.42 × 10^6^	6.04 × 10^19^
AD-NVO	340.02	1.30 × 10^6^	3.24 × 10^19^

The potential window of 1.20–3.50 V was selected for the electrochemical cycling of AD-NVO and MD-NVO electrodes in nonaqueous Na-ion cells to enable relatively high capacities and cycling stability (Figure S4 and the Supporting Information). In addition, the AD-NVO and MD-NVO powders were vacuum-dried at 200 °C, as specified in the experimental section, to minimize the interlayer water content and suppress parasitic processes caused by the interlayer water. According to the XRD patterns shown in Figure S5 (Supporting Information), vacuum-drying at 200 °C does not lead to phase transformation of the AD-NVO and MD-NVO powders. Figure 5 demonstrates the first, second, and fifth cycles of CV curves of the AD-NVO and MD-NVO electrodes in nonaqueous Na-ion cells at 0.1 mV s^–1^. The AD-NVO electrode shows rectangular-shaped CV curves without obvious redox peaks (Figure 5a), suggesting that the charge transport in AD-NVO may be dominated by capacitive-like processes. This behavior can be attributed to the charge-shielding of the interlayer water, which facilitates pseudocapacitive charge storage.^39^ In contrast, distinct redox peaks can be observed in the CV curves of MD-NVO electrode (Figure 5b), suggesting the strong Faradaic charge storage processes with diffusion-limited charge transport behavior.^39^ During the first discharge cycle, AD-NVO exhibits a major cathodic peak at 1.50 V, which shifted to 1.75 V during the subsequent discharge cycles, pairing with an anodic peak that appeared at 2.10 V during all charge cycles. Additionally, a few reversible redox peaks with smaller corresponding current densities at higher potentials (2.6–3.5 V) can be identified in the CV curves of the MD-NVO electrode (Figure 5b).

**Figure 5:**
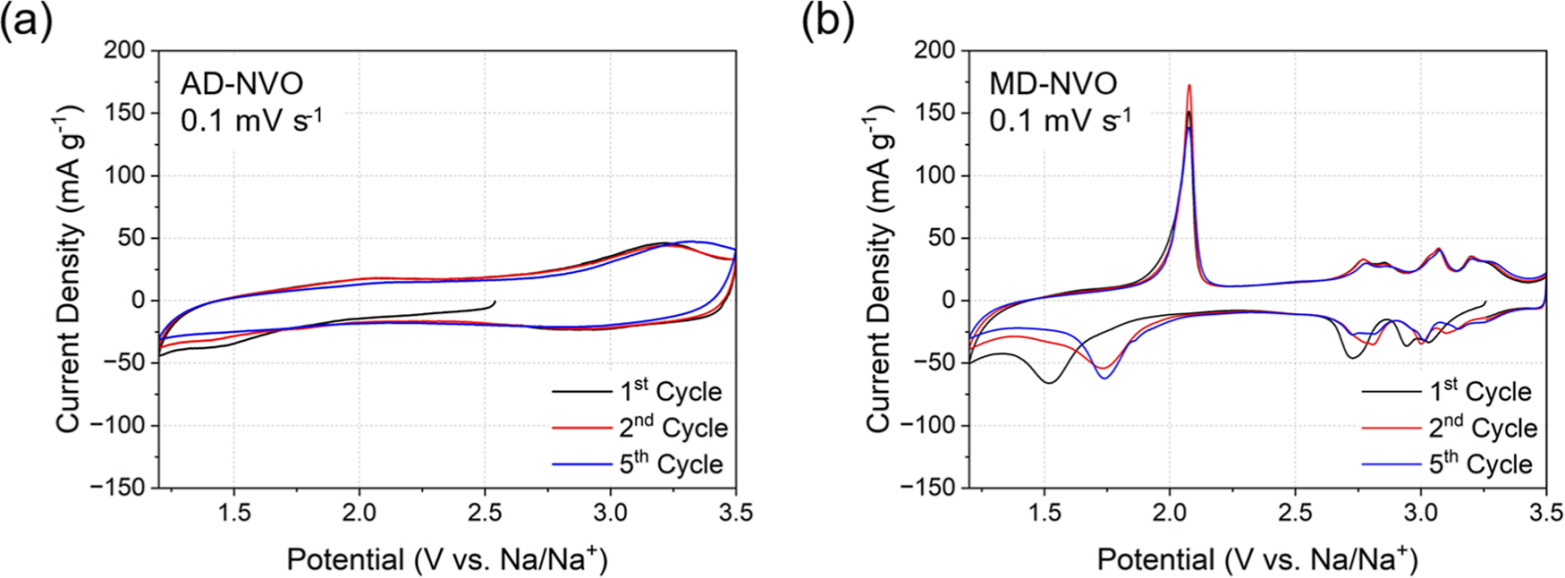
1st, 2nd, and 5th cycle CV curves at a sweep rate of 0.1 mV s^–1^ for the Na-ion cells containing (a) AD-NVO and (b) MD-NVO electrodes.

The galvanostatic discharge–charge (GDC) curves shown in Figure 6a,b indicate that the AD-NVO and MD-NVO electrodes delivered the initial specific capacities of 163.9 mAh g^–1^ and 170.4 mAh g^–1^, respectively, at a current density of 20 mA g^–1^ within a potential window of 1.20–3.50 V. The AD-NVO electrode exhibits nearly linear GDC profiles, consistent with the potential-independent charge-storage behavior indicated by its rectangular CV curves. In contrast, the MD-NVO electrode shows a distinct first-cycle discharge plateau, corresponding to Na^+^ ion intercalation, near ∼1.5 V, which shifts to ∼1.75 V in subsequent cycles. This process is reversed during charge, as indicated by the plateau at ∼2.10 V, consistent with the redox peak potentials observed in the CV curves of the MD-NVO electrode. Additionally, several smaller discharge–charge plateaus appear between 2.60 and 3.50 V, in good agreement with the redox features revealed by the CV analysis. In addition, upon the comparison of the GDC profiles for both electrodes at 50th and 100th cycles with corresponding profiles at initial cycles (1st, 2nd, and 5th cycles), we observed capacity degradation in both electrodes without a signal that could correspond to the charge storage mechanism evolution in either material over extended cycling: the AD-NVO electrodes maintained a linear-like potential–capacity profile, and the MD-NVO electrodes continued to exhibit charge–discharge plateaus at similar potentials in the 100th cycle relative to their respective initial cycles.

**Figure 6:**
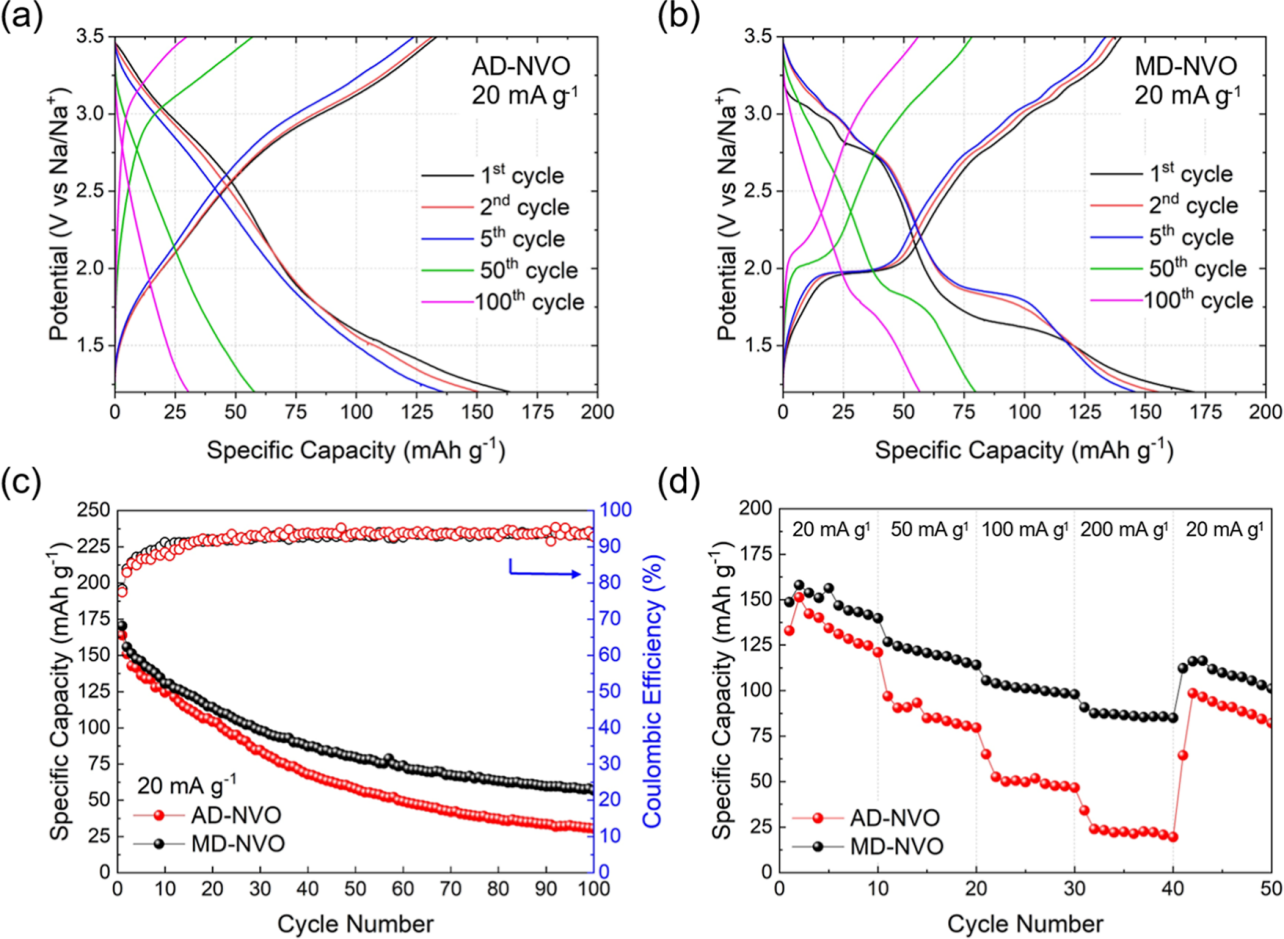
1st, 2nd, 5th, 50th, and 100th cycle GCD curves at a current density of 20 mA g^–1^ for (a) AD-NVO and (b) MD-NVO electrodes; comparison of (c) cycle life at a current density of 20 mA g^–1^ for 100 cycles, and (d) rate capabilities of AD-NVO and MD-NVO electrodes at 20, 50, 100, 200, and 20 mA g^–1^ each for 10 cycles in nonaqueous Na-ion cells.

The specific capacities and Coulombic efficiencies of AD-NVO and MD-NVO electrodes cycled for 100 galvanostatic cycles at a current density of 20 mA g^–1^ are shown and compared in Figure 6c. The first cycle Coulombic efficiency for AD-NVO and MD-NVO electrodes are 81.34% and 82.23%, respectively. The relatively low Coulombic efficiencies indicating the irreversible loss of Na inventory during initial cycles due to the formation of the fluorinated cathode-electrolyte interface^40^ can be the major contribution of the capacity degradation during the first 10 cycles. As the cycling continues, the Coulombic efficiencies for both AD-NVO and MD-NVO electrodes gradually increase and stabilize at around 98%, and the further capacity decay could be attributed to the structural degradation^14^ and parasitic processes caused by interlayer water.^13^ After 100 cycles, MD-NVO maintained a specific capacity of 56.8 mAh g^–1^, corresponding to 33.3% capacity retention. At the same time, AD-NVO delivered a capacity retention of only 30.6 mAh g^–1^ (18.7% capacity retention) after 100 cycles. We believe that due to a higher hydration degree of AD-NVO, as compared to MD-NVO, its interlayer structural water may be extracted from the interlayer region during cycling causing more pronounced structural degradation and deterioration of the water-sensitive cell components.

Figure 6d shows the rate capabilities of the AD-NVO and MD-NVO electrodes in Na-ion cells. The first 10 cycles of the rate capability test exhibited similar cycling characteristics for both electrodes as during their first 10 cycles in the cycle life test (Figure 6c). At the current densities of 50, 100, and 200 mA g^–1^, the AD-NVO electrode exhibited average specific capacities of 86.7, 51.0, and 23.3 mAh g^–1^, which are substantially lower than the 120.1, 101.2, and 86.7 mAh g^–1^ delivered by the MD-NVO electrode under the cycling conditions, respectively. After the current density was returned to 20 mA g^–1^ (cycles 41–50 in the rate capability experiments), the AD-NVO delivered an average specific capacity of 98.6 mAh g^–1^, which showed underperformance from the average capacity of 110.1 mAh g^–1^ delivered by the MD-NVO electrode under the same cycling condition. The higher specific capacities delivered by the MD-NVO electrode at all current densities indicate a relatively high electronic conductivity of MD-NVO, which could be attributed to a higher fraction of the V^4+^/V^5+^ mixed state in its V–O bilayers, as the V 2p XPS analysis (Figure 4) indicated.

To gain deeper insight into the structural features of Na-preintercalated NVO electrodes that influence their charge-storage behavior in Na-ion cells, we investigated the local environments of Na and V using solid-state MAS NMR. ^23^Na MAS NMR spectra of MD-NVO and AD-NVO are displayed in Figure 7a. The ^23^Na NMR spectrum of MD-NVO shows two distinct peaks at −32.81 and −55.24 ppm, suggesting two Na-sites, named site #1 and site #2, respectively. The ^23^Na NMR spectrum of AD-NVO showed a single peak appearing at −22.78 ppm, which implies that only one interlayer Na site exists in this material. It has been reported that metal ions residing in the interlayer structure of layered materials show negative chemical shifts in ss-NMR spectra, a feature attributed to the desolvation of the confined cations in the interlayer region.^28,29^ In the interlayer region of AD-NVO and MD-NVO, the Na-coordinating solvent is structural water, and negative shifts have been observed for all ^23^Na NMR spectra in this work, confirming the creation of the confined interlayer Na-sites via chemical preintercalation. For MD-NVO, the more negative ^23^Na resonance in site #2 suggests less structural water coordination than in site #1, and chemically preintercalated Na^+^ ions in both sites in MD-NVO (−32.81 and −55.24 ppm) appear to be less hydrated than the single Na-site identified in AD-NVO (−22.78 ppm). As a result, in MD-NVO, the Na^+^ ions occupying site #2 may engage in stronger electrostatic interactions with the V–O layers in the BVO framework, promoting a more pronounced V^5+^ → V^4+^ reduction, confirmed by the XPS results (Figure 4a,b), and forming additional interlayer bonds that enhance structural stability during electrochemical cycling. Meanwhile, the Na^+^ ions at site #1 may remain electrochemically active due to water-assisted ion transport, thereby facilitating Na^+^ diffusion.^11^ As the Na^+^ ions in site #2 of MD-NVO form stronger electrostatic bonds with the V–O bilayers, they may become electrochemically inactive and act as interlayer pillars, helping to prevent degradation of the layered structure during extended cycling. Additionally, we believe that the Na site #2 in MD-NVO enhances electronic conductivity by bridging the interlayer electron transport pathways and reducing a larger fraction of vanadium to the V^4+^ state, thereby leading to superior rate capabilities.^41^ In contrast, the NMR analysis suggests no such structure stabilizing Na-site in AD-NVO. Therefore, the AD-NVO electrode exhibited poorer cycling stability and rate capability, compared to the MD-NVO electrode, as well as demonstrated weak vanadium reduction, indicated by the XPS measurements (Figure 4a). The structure schematics of the AD-NVO and MD-NVO designed based on our analyses are shown in Figure S6 in the Supporting Information. By integrating the peaks in the ^23^Na NMR spectra, we determined that the ratio of total interlayer Na^+^ ions in MD-NVO and AD-NVO is 1.16, which is in good agreement with the ratio of 1.12 obtained via AAS analysis (Table 1). This observation confirmed that the different vanadium reduction intensities in both materials suggested by XPS analysis are most likely attributed to the different hydration degrees of interlayer Na^+^ ions, leading to the variation of interlayer Na sites with distinct chemical environment and structural characteristics.

**Figure 7:**
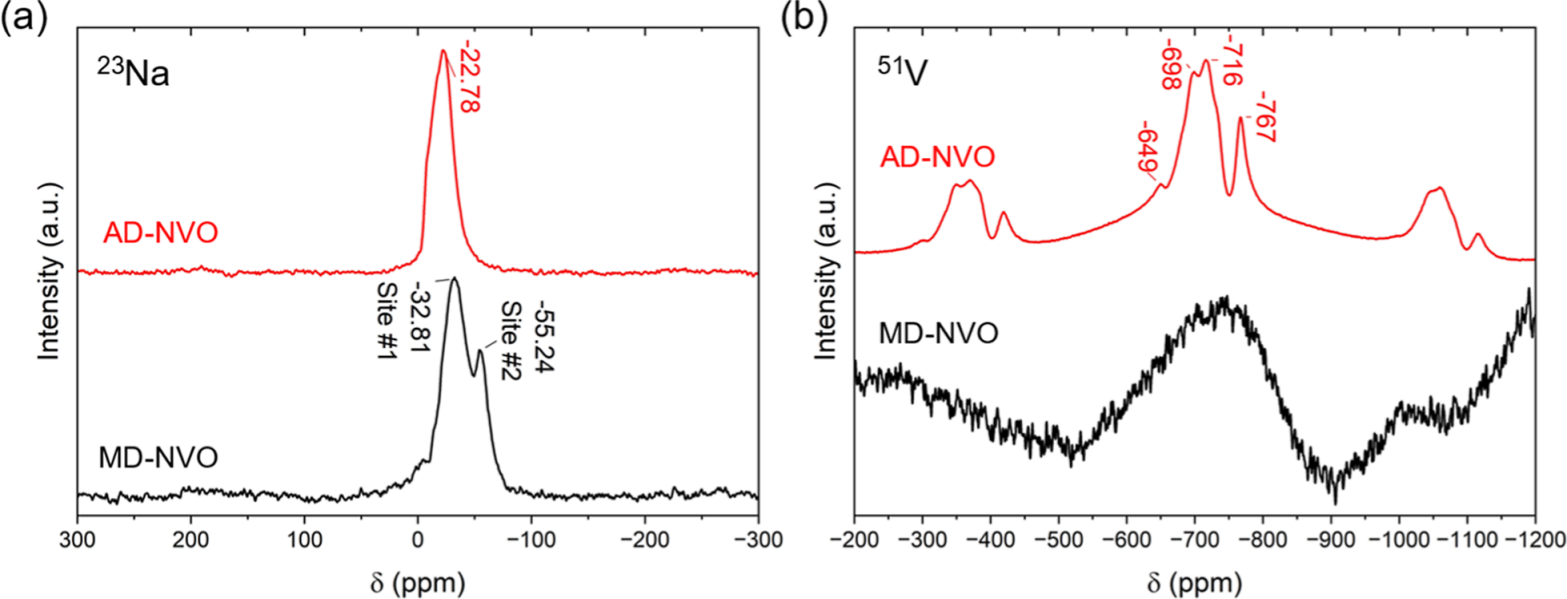
(a) ^23^Na and (b) ^51^V MAS ss-NMR spectra for AD-NVO (spin rates: 18 kHz) and MD-NVO (spin rates: 17 kHz) samples. ^23^Na and ^51^V measurements were performed at 7 and 14 T magnetic fields, respectively.

To verify our hypothesis that the formation of a second interlayer Na site in MD-NVO, revealed by ^23^Na ss-NMR, enhances cycling stability through a pillaring effect and structural stabilization, we performed postcycling ex situ XRD analysis of both electrodes after 100 cycles in Na-ion cells. As shown in Figure 8, the XRD patterns of the pristine MD-NVO and AD-NVO electrodes indicate a layered structure with *d*-spacings of 10.87 and 11.11 Å, respectively. The slight increase in *d*-spacing compared to the material powders (Figure 2a) is caused by the processing steps used to fabricate the electrodes. After 100 cycles at a current density of 100 mA g^–1^, the XRD pattern of the AD-NVO electrode shows significant peak broadening, indicating severe structural degradation, likely due to the absence of the pillaring Na site in the interlayer region, as revealed by ss-NMR characterization (Figure 7a). In contrast, the XRD pattern of the cycled MD-NVO electrode exhibits a distinct (001) peak corresponding to an interlayer distance of 8.91 Å, indicating that its layered structure is well-maintained during extended cell operation. This enhanced structural stability can be attributed to the presence of pillaring Na atoms with lower hydration and stronger bonding to the V–O bilayers (site #2 at −55.24 ppm), as suggested by ^23^Na ss-NMR spectra (Figure 7a). Overall, these results highlight the critical role of both the precursor and the interlayer Na sites in preserving the structural integrity of MD-NVO during prolonged electrochemical cycling, with the MXene precursor promoting the formation of pillaring Na atoms that stabilize the layered framework compared to the AD-NVO.

**Figure 8:**
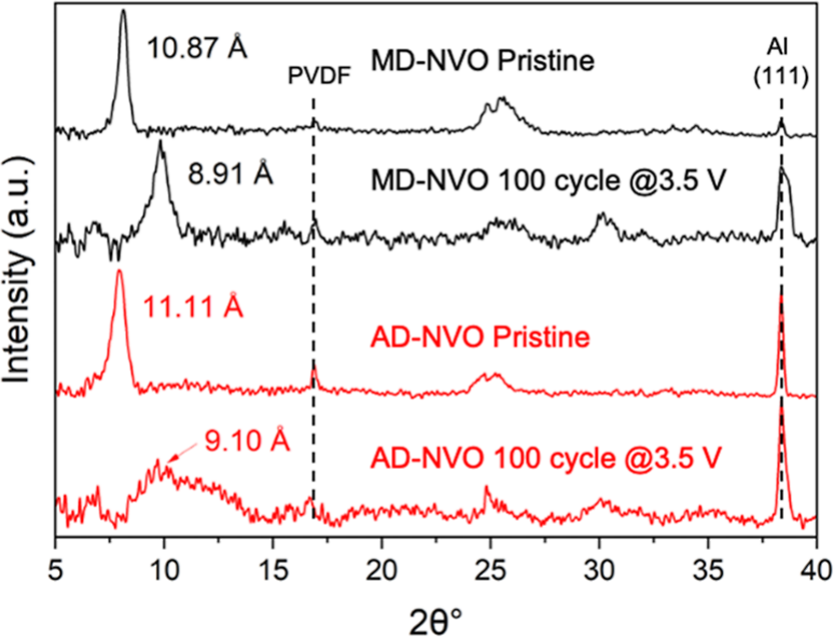
Ex situ XRD analysis of the pristine electrodes and the electrodes after 100 electrochemical discharge/charge cycles at 100 mA g^–1^.

^51^V MAS ss-NMR spectra of MD-NVO and AD-NVO are shown in Figure 7b. The ^51^V NMR spectrum for AD-NVO displays two closely located vanadium sites at −699 and −716 ppm and the smaller inequivalent sites at around −649 and −767 ppm with corresponding side bands. However, the ^51^V NMR spectrum for MD-NVO exhibited a signal virtually indistinguishable from noise (likely due to probe ringdown), attributed to the presence of a large fraction of paramagnetic V^4+^ ions, revealed by the XPS analysis, which are insensitive for the ss-NMR technique. To further confirm this observation, the paramagnetic V^4+^ content in both samples was quantified by EPR measurements (Figure S7, Supporting Information), and the spin quantifications are summarized in Table 2. These results confirm a higher fraction of V^4+^ ions in MD-NVO, in agreement with the XPS findings. Interestingly, EPR analysis indicates a higher number of V^4+^ spins in AD-NVO than that suggested by the V 2p XPS data (Figure 4a). Given the strong surface sensitivity of XPS, it is likely that the additional V^4+^ species detected by EPR reside in the bulk of AD-NVO and are not detectable by XPS. Nevertheless, the number of V^4+^ spins in MD-NVO remains nearly twice that in AD-NVO, emphasizing that the interlayer structural water content plays a more decisive role in the extent of vanadium reduction as the degree of Na preintercalation is similar in both materials. Apparently, the factor of 2 increase in spin density is sufficient to essentially suppress the NMR signal of the remaining V^5+^ in MD-NVO. A more sophisticated analysis of electron–nuclear spin coupling or the examination of additional samples with intermediate spin-density values may provide further insights; however, such an investigation is beyond the scope of this work.

Intrigued by these findings and to explore whether the locally observed structural discrepancies also occur in other metal-ion preintercalated BVO polymorphs, we investigated Li-preintercalated BVO phases derived from α-V_2_O_5_ powder (AD-LVO) and V_2_CT_*x*_ MXene nanoflakes (MD-LVO). As shown by ^7^Li MAS ss-NMR measurements (Figure S8, Supporting Information), the interlayer Li-site trends are similar to those observed for the NVO phases. The ^7^Li ss-NMR spectrum of AD-LVO exhibits a single peak at −3.0 ppm, whereas MD-LVO shows two distinct peaks at −1.9 and −10.0 ppm. This observation aligns with the previously reported enhanced cycling stability of MD-LVO^19^ relative to AD-LVO^23^ in Li-ion cells. Similar to MD-NVO, the chemically preintercalated Li^+^ ions corresponding to the more negative chemical shift (−10.0 ppm) in MD-LVO likely act as electrochemically inactive pillars, stabilizing the structure and improving the cyclability. These results further support the conclusion that the two-site enhancement mechanism revealed by ss-NMR may be generally applicable to other chemically preintercalated MXene-derived BVO electrodes, such as K-preintercalated bilayered vanadium oxide (MD-KVO) in a K-ion energy storage system.^19,31^

Our work highlights the compositional and structural differences in NVO electrode materials derived from α-V_2_O_5_ and V_2_CT_*x*_ MXene and correlates these features with their electrochemical cycling behavior in nonaqueous Na-ion cells. We demonstrated for the first time that by using V_2_CT_*x*_ MXene as a precursor for the synthesis NVO, the chemically preintercalated Na^+^ ions occupy two distinct sites in the interlayer region, one of which is characterized by a lower hydration degree than the other and plays a role in enhancing cycling stability (pillaring site), while the more highly hydrated Na site is electrochemically active and facilitates interlayer Na diffusion. The ex situ XRD analysis of the post-cycled NVO electrodes confirmed superior structural stability of the MD-NVO electrode after extended electrochemical cycling, further supporting the structure stabilizing effect enabled by the pillaring Na sites. This work for the first time shows that the single metal ion can be located at different sites in the interlayer structure by using different synthesis precursors, offering new insights into designing high-performance electrode materials for SIBs through a chemical preintercalation strategy.

## Conclusions

This work established mechanistic correlations between the precursor-enabled structural and compositional discrepancies in Na-preintercalated BVOs with their unique electrochemical cycling properties in nonaqueous Na-ion cells. Unlike previous studies focused on the evolution of interlayer distance and unique surface morphologies, we demonstrate for the first time that the chemically preintercalated interlayer metal-ion sites can play synergistic roles in enhancing specific capacity and rate capability and stabilizing the electrochemical cycling. By combining AAS and TGA analyses, we revealed the compositional differences between two materials enabled by synthesis precursors Na_0.37_V_2_O_5_·0.46H_2_O for AD-NVO and Na_0.33_V_2_O_5_·0.21H_2_O for MD-NVO. These compositional insights were further correlated with the discrepancies in the V^4+^ fractions, implying that the stronger vanadium reduction in MD-NVO was induced by the formation of additional interlayer bonds due to stronger Na-induced electrostatic interactions associated with the lower hydration degree. Most importantly, we have shown two distinct Na sites in the interlayer region of MD-NVO in contrast to the single Na site in AD-NVO. Our analyses revealed that one of the interlayer Na sites in MD-NVO enhances the cycling stability and rate capabilities by serving as an electrochemically inactive pillar and preserving the layered structure framework over extended cycling, as confirmed by ex situ XRD measurements. The structural characteristics of Na-preintercalated BVOs exhibit similarities to those of Li-preintercalated BVOs, suggesting the generalizable mechanisms governing chemical preintercalation and the enhancement of precursor-enabled electrochemical performance. Our findings open a pathway for exploring precursor-enabled structural and compositional characteristics in other metal-ion preintercalated layered metal oxides, providing further insights in advancing the electrochemical performance of layered electrode materials for intercalation batteries.

## Supplementary Material



## Data Availability

Data for this article are available at *Materials Commons 2.0* repository at https://doi.org/10.13011/m3-mma6-wn70.

## Notes

Synthesis schematics and structural model illustrations; AAS data and calibration curves; and supplementary XRD, XPS, CV, solid-state NMR, and EPR characterization results (PDF)
