# Supplementary material for: Solid-State Nuclear Magnetic Resonance Insights into the Precursor-Dependent Structure and Na-Ion Storage Behavior of Na-Preintercalated Bilayered Vanadium Oxides
Source: Chem Mater. 2026 Apr 24;38(9):4636–46. doi: 10.1021/acs.chemmater.6c00065 (PMC13173501; doi:10.1021/acs.chemmater.6c00065)
Supplement: Supplementary file 1 [file cm6c00065_si_001.pdf]

Supporting Information

Solid-State Nuclear Magnetic Resonance Insights into Precursor-Dependent Structure and Na-Ion Storage Behavior of Na-Preintercalated Bilayered Vanadium Oxides

Xinle Zhang[1], Timofey Averianov[1], Mina Mozafari[2], Phillip Stallworth[2], Dmitri Barbash[3], Steven G. Greenbaum[2], Ekaterina Pomerantseva[1*]

1 Department of Materials Science and Engineering, Drexel University, Philadelphia, PA 19104, USA

2 Department of Physics & Astronomy, Hunter College of CUNY, New York, NY 10065, USA

3 Materials Characterization Core Facilities, Drexel University, Philadelphia, PA 19104, USA

* Corresponding Author: ep423@drexel.edu

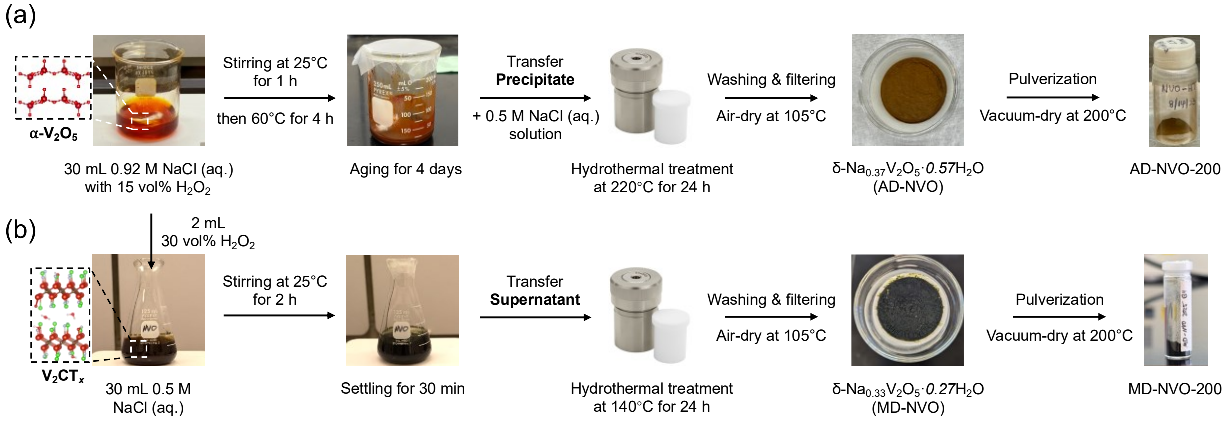

Figure S1. Illustrations of the material synthesis procedures for (a) AD-NVO and (b) MD-NVO.

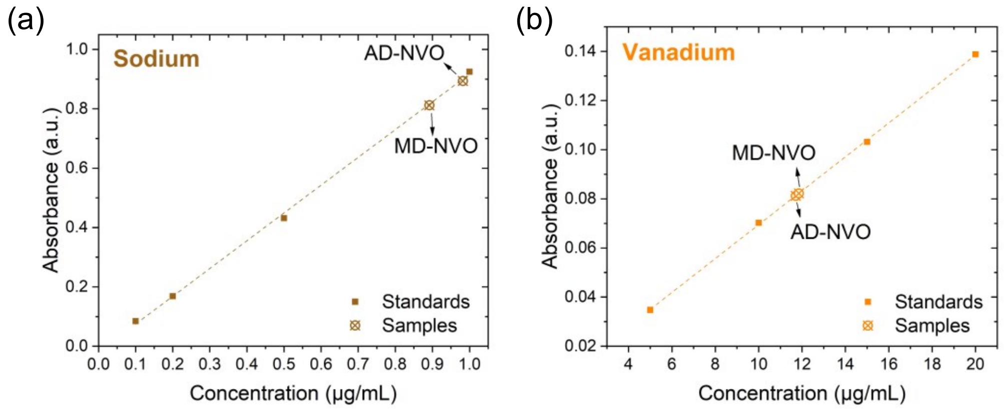

Figure S2. AAS calibration curves of (a) sodium and (b) vanadium, and corresponding concentrations in AD-NVO and MD-NVO samples in this study.

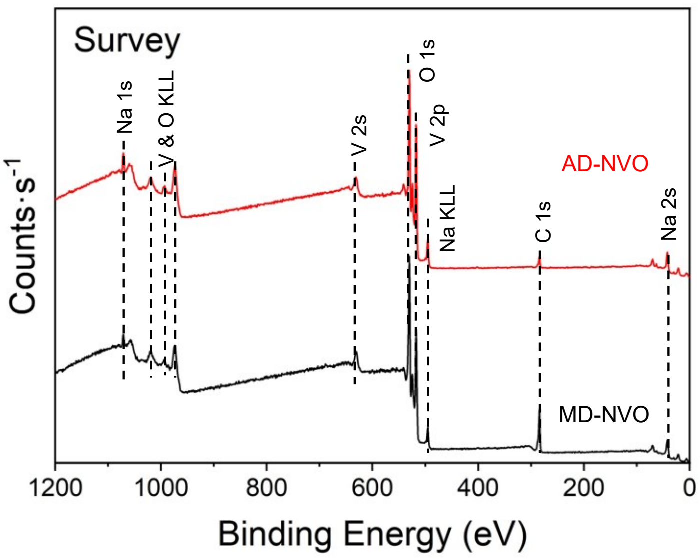

Figure S3. XPS survey spectra of AD-NVO and MD-NVO powders, showing the photoelectron signals from Na, V, and O.

We investigated the CV curves for the MD-NVO electrodes (without additional drying steps for active materials and electrodes) at multiple potential windows within a range of 1.0 – 4.0 V (vs. Na/Na+, also applies to the following potentials), shown in **Figure S4**, Supporting Information. Our investigation revealed that, within a relatively high potential window of 2.0-4.0 V, the CV curves includes a pair of redox peaks at 3.50 V (anodic) and 3.75 V (cathodic), which is believed to attribute the collapse of the layered structure due to the extraction of the interlayer Na^+ ions and structural water, leading to the rapid fade of the redox peaks within the initial cycles. As this pair of redox peaks was excluded by cutting the upper potential limit to 3.50 V, the redox peaks were retained better within the same initial cycles, and almost no fading can be observed when potential window was narrowed to 2.0 – 3.3 V. Upon considering the tradeoff of electrode's reversibility and specific capacity, we decided to proceed with the potential window of 1.20 – 3.50 V, which showed an acceptable redox reversibility and an initial discharge capacity of 182 mAh g^{-1}, for the following evaluation of the Na-ion cycling performance of the AD-NVO and MD-NVO electrodes.

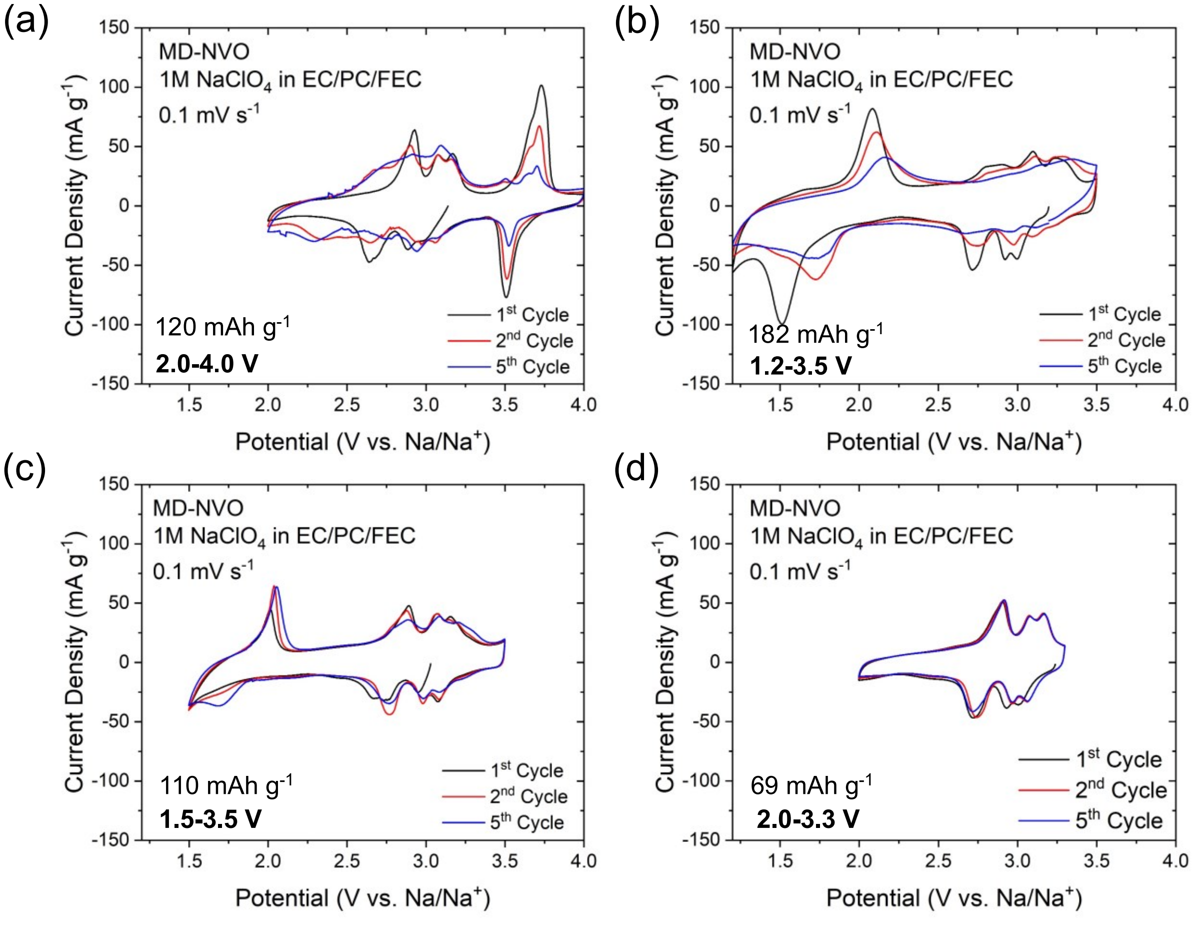

Figure S4. CV curves of MD-NVO electrodes in 1st, 2nd, and 5th cycles at 0.1 mV s^{-1} within (a) 2.0 – 4.0 V, (b) 1.2 – 3.5 V, (c) 1.5 – 3.5 V, and (d) 2.0 – 3.3 V, versus Na/Na^+, respectively.

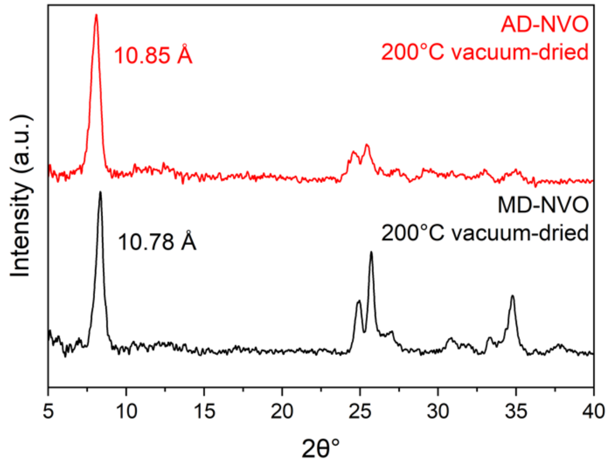

Figure S5. XRD patterns of AD-NVO and MD-NVO powder after vacuum-drying at 200°C for 48 hours show no substantial change in layered structure and interlayer distance.

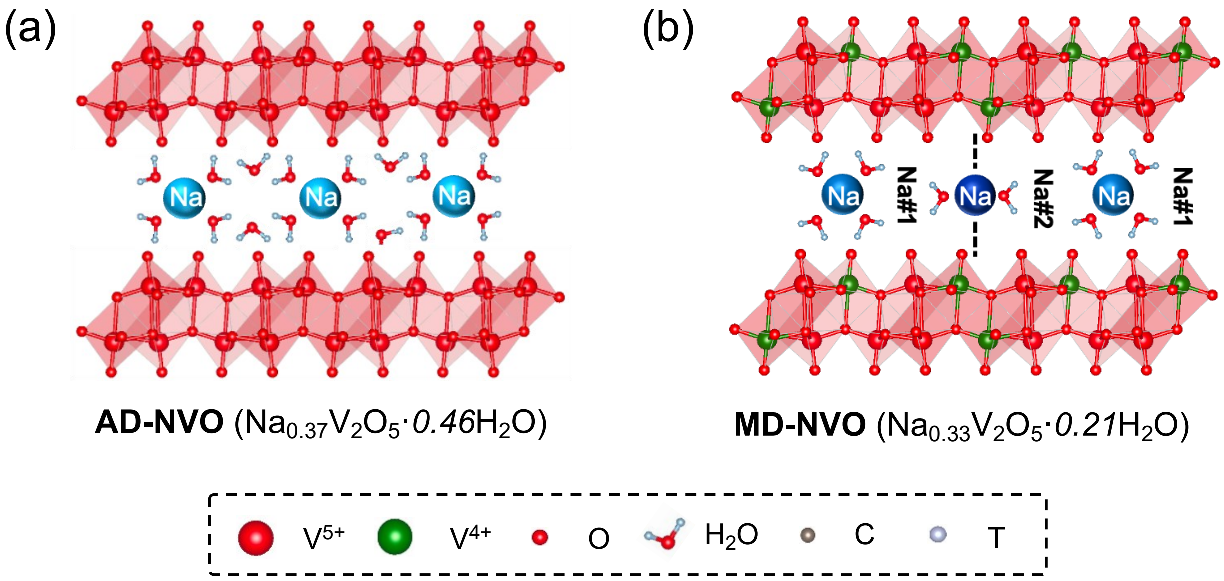

Figure S6. Structure schematics based on the ^{23}Na ss-NMR and V 2p XPS analyses showing the structures of (a) AD-NVO with single highly hydrated interlayer Na site that correspond to the -22.78 ppm peak in ^{23}Na ss-NMR spectra, and (b) MD-NVO with site #1 and #2 corresponding to the -32.81 and -55.24 ppm ^{23}Na ss-NMR peaks, respectively, with additional bonds illustrated by thick black dash lines next to the Na site #2, which led to the V^{4+}/V^{5+} mixed valance states represented by alternative green/red atoms within the bilayers.

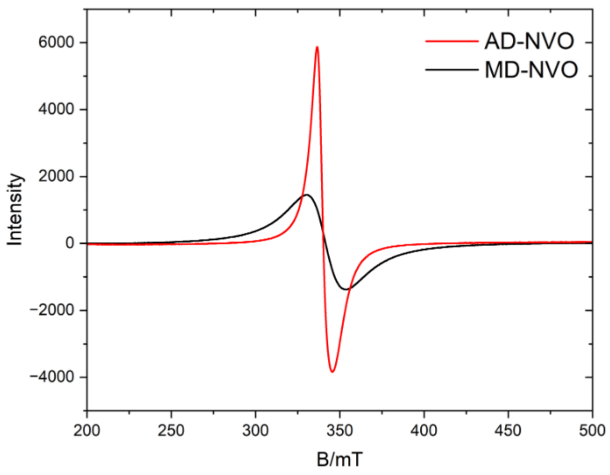

Figure S7. EPR spectra of AD-NVO and MD-NVO were used for quantifying the V^{4+} spins.

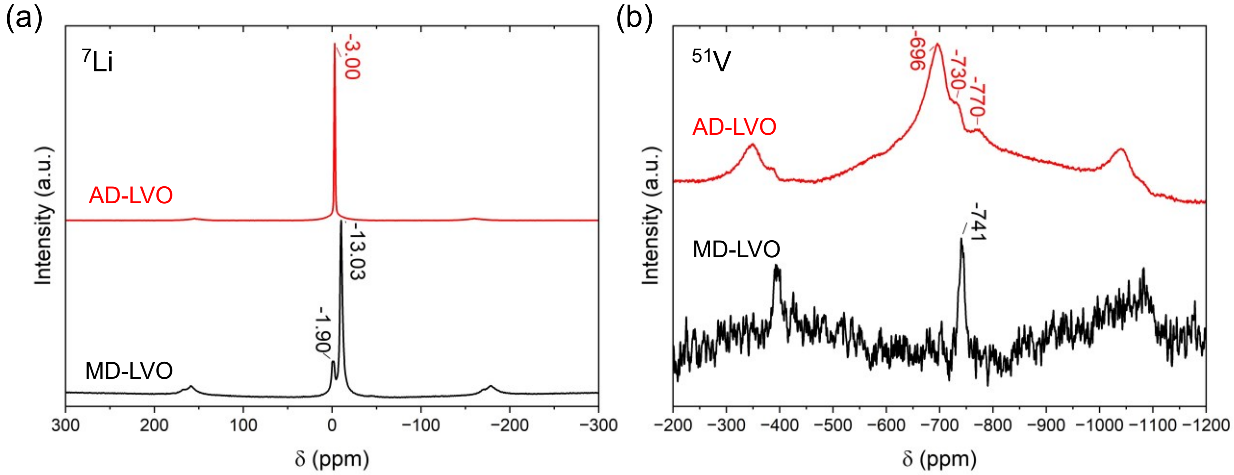

Figure S8. (a) ^{7}Li and (b) ^{51}V ss-NMR spectra for AD-LVO and MD-LVO samples.
